# The Senior Editor

**Published:** 1873-08

**Authors:** 


The Senior Editor is deeply grateful for the many kind notices in the secular press of his address delivered at the late commencement of the Wesleyan Female College, Staunton, Virginia. While undeserving of these encomiums, he nevertheless appreciates the kind and flattering opinions of his brethren of the "quill." His thanks are especially due the following papers: *Raleigh News*, North Carolina; *Rural Messenger*, Petersburg, Virginia; *Daily Index*, Petersburg, Virginia; *Columbus Democrat*, Columbus, Mississippi; *Daily Appeal*, Petersburg, Virginia; *The Valley Virginian*, Staunton, Virginia.

